# Case Report: A rare case of ANCA-positive Q fever endocarditis-associated glomerulonephritis

**DOI:** 10.3389/fmed.2026.1701814

**Published:** 2026-02-10

**Authors:** Qian Li, Suwen Liu, Jing Wang, Lichun Yu, Shuzhen Sun

**Affiliations:** Department of Pediatric Nephrology and Rheumatism and Immunology, Shandong Provincial Hospital Affiliated to Shandong First Medical University, Jinan, China

**Keywords:** antineutrophil cytoplasmic antibody, child, endocarditis, glomerulonephritis, Q fever

## Abstract

**Background:**

Q fever is a zoonotic disease caused by *Coxiella burnetii* and is endemic worldwide. Q fever endocarditis is commonly found in adults and is rarely seen in children. Infectious endocarditis can also lead to renal damage. Here, we present a case of Q fever endocarditis-associated glomerulonephritis in a Chinese boy with positive Antiproteinase 3 antibody (PR3)-antineutrophil cytoplasmic antibody (ANCA).

**Case presentation:**

A 12-year-old Chinese boy presented with intermittent fever and hematuria for 2 months. He was diagnosed with Tetralogy of Fallot at birth and underwent multiple cardiac surgeries between 1 and 4 years of age. The examinations showed positive serum mycoplasma antibody and increased serum Epstein–Barr virus (EBV) DNA. However, antibiotic and antiviral treatment was not effective. PR3-ANCA antibody was positive (109.8–158.8CU), while anti-myeloperoxidase (MPO) antibody, anti-glomerular basement membrane (GBM) antibody, antinuclear antibodies (ANA), and double-stranded DNA (dsDNA) were negative. Chest CT showed bronchitis. Ophthalmic examination and ENT examinations revealed no abnormalities. *Coxiella burnetii* was found positive by metagenomics next generation sequencing (mNGS) and immunofluorescence assay (IFA) in the detection of pathogenic microorganisms causing bloodstream infections. Prominent vegetation was present on the pulmonary valve, as demonstrated by cardiac ultrasound. Secondary hyperplastic glomerulonephritis was considered by renal biopsy. Therefore, the final diagnosis was Q fever endocarditis-associated glomerulonephritis. Doxycycline was given to the boy orally and daily, and no fever occurred again. Sixteen months later, hematuria disappeared and PR3-ANCA remainded positive.

**Conclusions:**

Q fever endocarditis should be considered for children presenting with chronic fever, hematuria and positive ANCAs, especially those with a history of congenital heart disease or cardiac operation. It is very helpful for the diagnosis to undergo these examinations, including mNGS, cardiac ultrasound and renal biopsy.

## Introduction

Q fever is a zoonotic disease caused by *Coxiella burnetii* and is endemic worldwide (1). It can be divided into asymptomatic, acute, and chronic infections. Approximately 40%−60% of *Coxiella burnetii* infections have no clinical symptoms (2), and 1.0%−5.0% of acute infections may progress to persistent infections (3). Patients with Q fever can present with pneumonia, encephalitis, meningitis, osteomyelitis, hepatitis, pericarditis, endocarditis, hemophagocytic syndrome, alithiasic cholecystitis, and isolated fever and/or flulike syndrome (4, 5). Q fever endocarditis is commonly found in adults (6) and is rarely seen in children. Infectious endocarditis can also lead to renal damage. Here, we present a rare case of Q fever endocarditis-associated glomerulonephritis in a Chinese boy with positive Antiproteinase 3 antibody (PR3)-antineutrophil cytoplasmic antibody (ANCA).

## Case description

A 12-year-old boy was admitted to our hospital with complaints of intermittent fever and hematuria for 2 months. The child had been examined before admission to our hospital. The examinations in a local hospital showed mild hematuria, positive serum mycoplasma antibody, increased serum Epstein–Barr virus (EBV) DNA, positive PR3-ANCA, an increased erythrocyte sedimentation rate (ESR), and nutcracker syndrome by ultrasound (Table 1). This patient was diagnosed with acute nephritis, mycoplasma infection, EBV infection, hepatic damage, nutcracker syndrome and suspected ANCA-associated vasculitis. He received anti-infection treatment with melloxicillin, cephalosporin, azithromycin, and acyclovir successively. However, the boy still presented with intermittent fever, accompanied by gross hematuria when he was febrile and microscopic hematuria when his temperature was normal.

**Table 1:** Laboratory results of the patient.

**Parameters**	**2 months ago (at the onset)**	**1.5 months ago**	**Time 0 (Admission in our hospital)**	**1 months later**	**16 months later**
White blood cells (10^9^/L)	7.6	5.9	5.69	4.02	4.0
Hemoglobin (g/L)	124	110	111	101	128
Platelets (10^9^/L)	190	156	119	193	197
AST (U/L)	163.2	208.7	147	37	27.5
ALT (U/L)	115.12	94	92	19	19.4
Creatinine (μmol/L)	43.5	47.8	49.5	56.3	33.1
eGFR (ml/min/1.73 m^2^)	120.8	109.9	106.2	93.4	165.4
C-reactive protein (mg/L)	6.6	16.5	6.73	8.0	ND
Erythrocyte sedimentation rate (mm/h)	ND	31	22	ND	2
Procalcitonin (ng/ml)	0.23	0.28	0.19	0.27	ND
Complement 3 (g/L)	ND	1.01	0.97	0.92	1.3
Complement 4 (g/L)	ND	0.147	0.18	0.18	0.157
Anti-PR3-ANCA antibody (RU/ml)	ND	>400	114.8	158.8	149.61
Anti-myeloperoxidase antibody (RU/ml)	ND	5.28	0.80	0.7	< 2.0
Anti-glomerular basement membrane antibody	ND	Negative	Negative	Negative	Negative
Anti-nuclear antibodies (ANA)	ND	Negative	Negative	ND	ND
Anti-double-stranded DNA (dsDNA)	ND	Negative	Negative	ND	ND
Rheumatoid factor (IU/ml)	ND	63.77	72.36	ND	11.76
**Urinalysis**					
Protein	(–)	(–)	(–)	(+)	(–)
Red blood cells (/HPF)	43.61	20.6	79.9	312.6	0.89
Urinary protein/creatinine ratio (mg/mg)	ND	ND	0.30	0.72	ND
Urinary calcium/creatinine ratio (mg/mg)	ND	ND	0.01	ND	ND
24-h urine protein quantity (g)	ND	ND	0.29	0.57	0.1

AST, aspartate aminotransferase; ALT, alanine aminotransferase; eGFR, estimated glomerularfiltrationrate; ND, no data.

The boy was diagnosed with Tetralogy of Fallot at birth. When he was between 1 and 4 years of age, the patient underwent multiple cardiac surgeries, including palliative right ventricular pulmonary artery connection plus pulmonary angioplasty, percutaneous pulmonary artery balloon expansion, percutaneous pulmonary artery stent implantation, pulmonary collateral occlusion plus pulmonary artery stent balloon dilatation, and pulmonary atresia plus pulmonary angioplasty plus right ventricular outflow channel dredging plus patent foramen ovale repair. The last operation was performed 8 years prior. There was no family history. Physical examination revealed a loud cardiac systolic murmur and hepatosplenomegaly.

The patient underwent a series of examinations after admission. The urine tests showed protein (–) ~ (+), microalbumin 4.88–138 mg/L, red blood cells (RBCs) 24.2–366/HPF, and white blood cells (WBCs) 0.8–9.1/HPF. The urinary protein/creatinine ratio ranged from 0.30 to 0.71 mg/mg. The 24-h urine protein quantity was 0.29–0.57 g. Routine blood tests showed WBC 3.5–8.3 × 10^9^/L, RBC 16–4.79 × 10^12^/L, hemoglobin (Hb) 94–111 g/L, and platelets (PLT) 116–225 × 10^9^/L. The ESR was increased (19–22 mm/h), while procalcitonin was elevated (0.10–0.44 ng/ml). The C-reactive protein level was normal (Table 1). Blood cultures were negative. Pathogenic microorganisms that can cause bloodstream infections were detected by metagenomics next generation sequencing (mNGS), and the final result showed *Coxiella burnetii*, which can cause Query fever (Q fever). The relevant antibody examination showed positive *Coxiella burnetii* phase I IgG antibody and phase II IgG antibody by immunofluorescence assay (IFA). Serum complement was normal. PR3-ANCA antibody was positive (109.8–158.8CU), while anti-myeloperoxidase (MPO) antibody, anti-glomerular basement membrane antibody, antinuclear antibodies (ANA), and double-stranded DNA (dsDNA) were negative (Table 1). Chest CT showed bronchitis. Ophthalmic examination and ENT examinations revealed no abnormalities.

**Figure 1:**
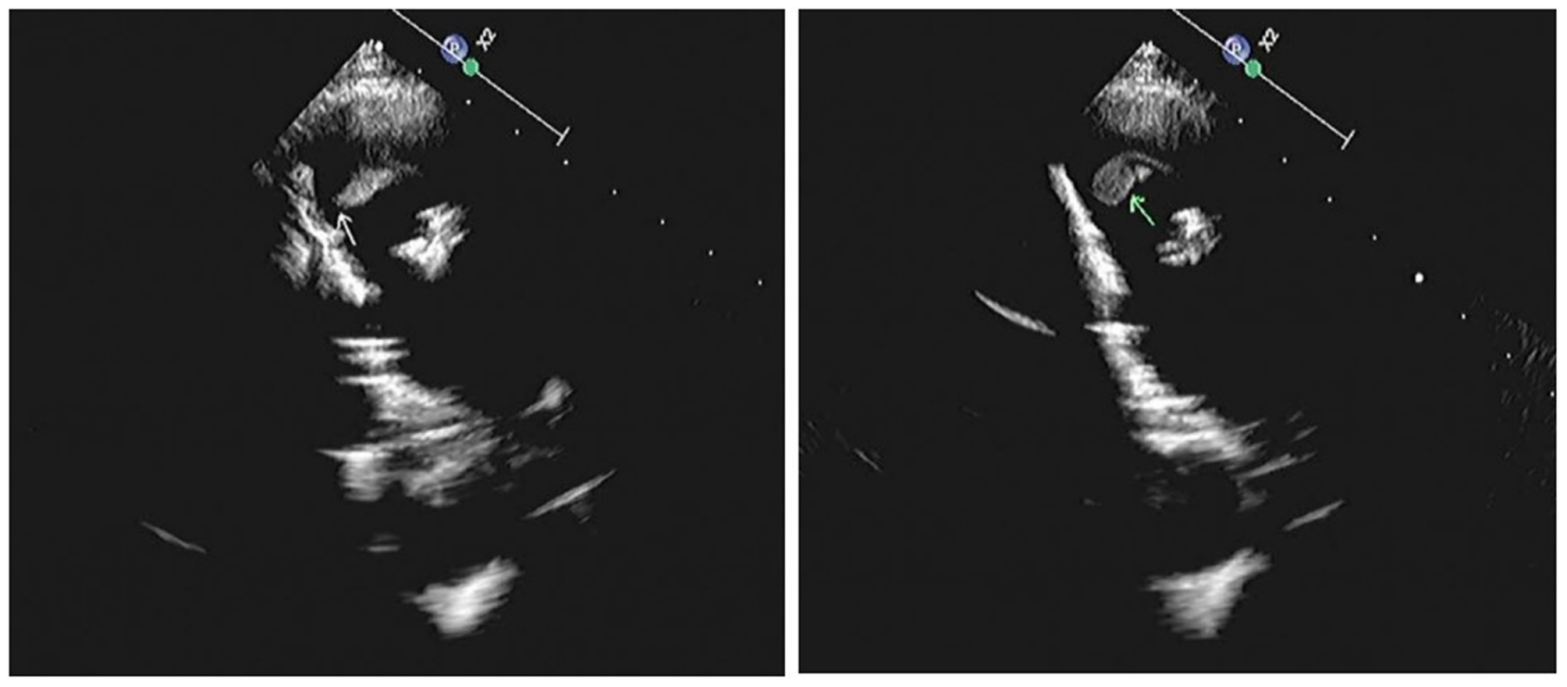
Cardiac ultrasound demonstrated pulmonary valve vegetation (arrow).

Cardiac ultrasound was applied. The enlarged right atrium and right ventricle and slightly widened ascending aorta are shown. The upper septum of the interventricular septum was found to have an echo of the patch (due to the previous cardiac surgeries). No echo of the vegetation was detected around the patch or in the tricuspid valve area. The echo of the valve-bearing artificial vessel in the pulmonary artery area was explored. The echo of the stent was observed in the pulmonary artery lumen, and the position was fixed. The pulmonary valve leaflets were thickened where the echo of the vegetation was detected, and the echo of the annulus was enhanced (Figure 1). The morphology and echo of the remaining valves were normal. Several body-pulmonary collaterals were observed in the descending part of the aortic arch. Prominent vegetation was present on the pulmonary valve.

Kidney biopsy was performed to observe the renal pathology. Light microscopy showed nineteen glomeruli. Two glomeruli were completely fibrotic. Mild diffuse and moderate segmental mesangial proliferation and segmental hyperplasia of endotheliocytes were observed. The capillary wall was slightly thickened. No definite small vasculitis changes were observed. Granular and vacuolar degenerations of renal tubular epithelial cells are shown. There were no obvious abnormalities in the walls of the small arteries. Immunofluorescence (IF) showed IgG ++, IgA +, C3+++, FRA +, IgM+++, C1q +++, κ +++ and λ +++, with a granular fluorescence distribution along the capillary wall. Electron microscopy showed that high-density massive electron-dense material deposits were observed in the subendothelial and mesangial areas. There were segmental foot process fusion in podocytes. Therefore, secondary hyperplastic glomerulonephritis was considered.

Therefore, the final diagnosis was Q fever endocarditis-associated glomerulonephritis. Doxycycline was given to the boy orally and daily, and no fever occurred again. Sixteen months later, hematuria disappeared and PR3-ANCA remainded positive (Table 1). The patient was recheck positive *Coxiella burnetii* phase I IgG antibody and phase II IgG antibody and continued to receive doxycycline orally. Long-term prognosis requires further follow-up to be determined.

## Discussion

Q fever endocarditis is a relatively rare disease that accounts for 5% of blood culture-negative infective endocarditis. Han et al. (7) reported six cases of Q fever endocarditis (4.08%) among 147 cases of blood culture-negative infective endocarditis in mainland China. The clinical manifestations of Q fever endocarditis are similar to those of common bacterial infective endocarditis, such as fever, heart murmur and weight loss, and valve dysfunction, heart failure and cerebral artery embolism can occur in severe cases. Unlike patients who have other causes of endocarditis, patients with Q fever endocarditis had a relatively mild inflammatory response and higher RF levels and were more likely to have biochemical indicators of other organ dysfunction, such as liver and kidney dysfunction (8).

The serological diagnosis of Q fever relies on the I phase and phase 2 antigens of *Coxiella burnetii*. Serology was positive in almost all cases and remains important in establishing a diagnosis of Q fever endocarditis (9). Immunofluorescence assay (IFA) is the preferred serological diagnostic technique for the diagnosis of Q fever due to its simple operation and high accuracy (10). Recently, there have been many reports on the discovery of *Coxiella burnetii* using mNGS technology (11). In this case, *Coxiella burnetii* was detected from the peripheral blood using mNGS, and the results of mNGS were subsequently confirmed by IFA.

Endocarditis usually occurs in patients who have prior valvular damage or who are immunocompromised (12). In patients undergoing valve replacement, the risk of infective endocarditis was significantly higher in patients with biological valves than in patients with mechanical valves (13). Deyell et al. (14) reported that transthoracic echocardiography is of limited value in Q fever diagnosis since vegetation is small or absent. However, in our report, this patient had a history of Tetralogy of Fallot and underwent multiple cardiac surgeries. Prominent vegetation was present on the pulmonary valve, and the findings by cardiac ultrasound aided in the diagnosis of Q fever endocarditis.

Infectious endocarditis can also lead to renal damage. The pathogenesis includes immune-mediated, direct infection of the pathogen, renal abscess, renal embolism, and vasculitis, so the clinical manifestations are complex. Activation of the complement system through the classical pathway is an important pathogenic mechanism that can cause a decrease in complement C3 and C4. Immunofluorescence examination of renal tissue showed glomerular C3 and other immunoglobulin deposits (15). Antinuclear antibody, rheumatoid factor, antiphospholipid antibody, ANCA and cryoglobulin can be positive (16). The clinical manifestations cover various types of glomerular disease, including asymptomatic hematuria or proteinuria, acute nephritis syndrome, nephrotic syndrome, and even aggressive glomerulonephritis. The renal pathology can be mesangial proliferative glomerulonephritis, membranogenic glomerulonephritis, crescent glomerulonephritis (17), renal infarction caused by renal vascular embolism, and focal nephritis caused by local renal embolism (18).

ANCAs are autoantibodies detected as substrates with normal human neutrophils and are classified into cytoplasmic ANCAs (c-ANCAs) and perinuclear ANCAs (p-ANCAs). The main target antigen of c-ANCA is proteinase 3 (PR3), while the main target antigen of p-ANCA is myeloperoxidase (MPO). ANCA is a specific serological marker of systemic vasculitis-associated diseases, also seen in non-vascular inflammatory diseases and infectious diseases, so positive ANCAs have been found in multiple infectious diseases. In recent years, with successive reports of cases of infective endocarditis with positive ANCAs (19), the relationship between infective endocarditis and ANCAs has received increasing attention.

The clinical manifestations of infective endocarditis can overlap with those of ANCA-associated vasculitis, such as fever, renal damage, lung damage, cutaneous purpura or ecchymosis, and splenomegaly. This patient presented with fever, hematuria, proteinuria, and positive ANCAs; therefore, he was more likely to be misdiagnosed with ANCA-associated glomerulonephritis. Kidney disease caused by infection usually belongs to immune complex deposition glomerulonephritis, while ANCA-associated glomerulonephritis shows oligoimmune complex or crescent formation. Therefore, renal biopsy is crucial to distinguish infection-associated glomerulonephritis and ANCA-associated glomerulonephritis. In this case, renal biopsy proved secondary proliferative glomerulonephritis and excluded ANCA-related glomerulonephritis.

Patients with infection-associated glomerulonephritis due to infective endocarditis should be given effective antibiotics and even need combined surgical treatment for heart valve destruction. Patients with acute renal impairment caused by infective endocarditis with positive ANCAs have a good prognosis after effective antibiotic treatment, suggesting that antibiotic treatment in these patients is the key and basic treatment. For patients with more cellular crescents, hormone therapy can be used to prevent the formation of new crescents and the transformation of cellular crescents into fibrous crescents. However, effective anti-infection therapy or valve replacement to remove infection are the most important for patients with fibrous crescents (20). Our patient received doxycycline treatment, and no fever or gross hematuria occurred again. One and a half months later, the boy still had microscopic hematuria and continued to receive doxycycline orally. The expected time of doxycycline therapy was 18 months and further follow-up and surveillance was needed.

The specific combination of pediatric Q fever endocarditis, glomerulonephritis, and PR-3 ANCA positivity presents diagnostic challenges or warrants particular attention. Pediatric Q fever endocarditis itself is inherently difficult to diagnose due to its atypical symptoms, which can easily be confused with other pediatric conditions. The presence of glomerulonephritis further complicates the clinical picture, potentially affecting renal function in affected children, necessitating precise differential diagnosis and timely, effective treatment. The positivity of PR-3 ANCA adds uncertainty to this combination, as it may be associated with multiple autoimmune diseases, thereby complicating the etiology, pathogenesis, and prognostic evaluation of the entire condition. This complex scenario places high demands on clinicians' diagnostic and therapeutic capabilities, making it a topic of special interest. In-depth investigation into its underlying mechanisms and pathophysiological relationships holds significant importance for advancing the clinical management of pediatric-related diseases.

In conclusion, Q fever endocarditis should be considered for children presenting with chronic fever, hematuria, proteinuria and positive ANCAs, especially those with a history of congenital heart disease or cardiac operations. It is very helpful for the diagnosis to undergo these examinations, including mNGS, cardiac ultrasound and renal biopsy.

## Data Availability

The original contributions presented in the study are included in the article/supplementary material, further inquiries can be directed to the corresponding authors.

## References

[1] Melenotte C Million M Raoult D. New insights in *Coxiella burnetii* infection: diagnosis and therapeutic update. Expert Rev Anti Infect Ther. (2020) 18:75–86. doi: 10.1080/14787210.2020.1699055 31782315

[2] Bacci S Villumsen S Valentiner-Branth P Smith B Krogfelt KA Mølbak K. Epidemiology and clinical features of human infection with *Coxiella burnetii* in Denmark during 2006-07. Zoonoses Public Health. (2012) 59:61–8. doi: 10.1111/j.1863-2378.2011.01419.x 21824371

[3] Kampschreur LM Oosterheert JJ de Vries Feyens CA Delsing CE Hermans MH van Sluisveld IL . Chronic Q fever-related dual-pathogen endocarditis: case series of three patients. J Clin Microbiol. (2011) 49:1692–4. doi: 10.1128/JCM.02596-10 21289146 PMC3122851

[4] Melenotte C Protopopescu C Million M Edouard S Carrieri MP Eldin C . Clinical features and complications of *Coxiella burnetii* infections from the French National Reference Center for Q fever. JAMA Netw Open. (2018) 1:e181580. doi: 10.1001/jamanetworkopen.2018.1580 30646123 PMC6324270

[5] Elzein FE Alsherbeeni N Alnajashi K Alsufyani E Akhtar MY Albalawi R . Ten-year experience of Q fever endocarditis in a tertiary cardiac center in Saudi Arabia. Int J Infect Dis. (2019) 88:21–6. doi: 10.1016/j.ijid.2019.07.035 31382048

[6] Balasubramanian R Fournier PE Ganesan PS Menon T. Q fever endocarditis in India: a report of two cases. Indian J Med Microbiol. (2022) 40:315–6. doi: 10.1016/j.ijmmb.2022.01.009 35153100

[7] Han X Hsu J Miao Q Zhou BT Fan HW Xiong XL . Retrospective examination of Q fever endocarditis: an underdiagnosed disease in the mainland of China. Chin Med J. (2017) 130:64–70. doi: 10.4103/0366-6999.196566 28051025 PMC5221114

[8] Zhang X Fan H Jiao Y Huang X. Defining the clinical characteristics of Q fever endocarditis: a case-control study in China. J Infect Dev Ctries. (2022) 16:1329–35. doi: 10.3855/jidc.15861 36099377

[9] Jaltotage B Ali U Dorai-Raj A Rankin J Sanfilippo F Dwivedi G. Q Fever endocarditis: a review of local and all reported cases in the literature. Heart Lung Circ. (2021) 30:1509–15. doi: 10.1016/j.hlc.2021.04.022 34052129

[10] Miller HK Binder AM Peterson A Theel ES Volpe JM Couturier MR . Trends in Q fever serologic testing by immunofluorescence from four large reference laboratories in the United States, 2012-2016. Sci Rep. (2018) 8:16670. doi: 10.1038/s41598-018-34702-2 30420599 PMC6232148

[11] Fu J Pi S Chen Y. Two case of Q fever endocarditis in Shenzhen city. Chin J Infect Dis. (2023) 41:538–9.

[12] Raizada A Apte N Pham S. Q fever endocarditis presenting with superior mesenteric artery embolism and renal infarction. Tex Heart Inst J. (2016) 43:91–3. doi: 10.14503/THIJ-14-4781 27047296 PMC4810597

[13] Mills MT Al-Mohammad A Warriner DR. Changes and advances in the field of infective endocarditis. Br J Hosp Med. (2022) 83:1–11. doi: 10.12968/hmed.2021.0510 35377207

[14] Deyell MW Chiu B Ross DB Alvarez N. Q fever endocarditis: a case report and review of the literature. Can J Cardiol. (2006) 22:781–5. doi: 10.1016/S0828-282X(06)70295-1 16835673 PMC2560519

[15] Chowdhury L Alobaidi A Lytvak I. Endocarditis-associated C3-dominant glomerulonephritis in a patient with a solitary kidney. Cureus. (2022) 14:e27675. doi: 10.7759/cureus.27675 35935112 PMC9351630

[16] Lefebvre M Grossi O Agard C Perret C Le Pape P Raoult D . Systemic immune presentations of *Coxiella burnetii* infection (Q Fever). Semin Arthritis Rheum. (2010) 39:405–9. doi: 10.1016/j.semarthrit.2008.10.004 19110298

[17] Miyata E Nakayama M Amano K Hirano T Uesugi N. A case of infectious endocarditis-associated crescentic glomerulonephritis with intracranial hemorrhage. J Nephrol. (2010) 23:738–42. 20155718

[18] Gao R Wen Y Li H Li X. Renal lesion associated with infectious endocarditis. Chin J Nephrol. (2005) 21:438–42.

[19] Zhang W Zhang H Wu D Fu H Shi W Xue F. Antineutrophil cytoplasmic antibody-positive infective endocarditis complicated by acute kidney injury: a case report and literature review. J Int Med Res. (2020) 48:300060520963990. doi: 10.1177/0300060520963990 33078666 PMC7583404

[20] Wang Z Jiang B Li X Zhen J Yang X Hu Z . A case of infective endocarditis and acute kidney injury with positive PR3-ANCA. J Shandong Univ. (2022) 60:60–4. doi: 10.6040/j.issn.1671-7554.0.2021.0836

